# Diversity of Perceptions of Disability in the Workplace vs. Cultural Determinants in Selected European Countries

**DOI:** 10.3390/ijerph19042058

**Published:** 2022-02-12

**Authors:** Dorota Kwiatkowska-Ciotucha, Urszula Załuska, Cyprian Kozyra, Alicja Grześkowiak, Marzena Żurawicka, Krzysztof Polak

**Affiliations:** 1 Department of Logistics, Wroclaw University of Economics and Business, 53-345 Wrocław, Poland; urszula.zaluska@ue.wroc.pl; 2 Department of Statistics, Wroclaw University of Economics and Business, 53-345 Wrocław, Poland; cyprian.kozyra@ue.wroc.pl; 3 Department of Econometrics and Operational Research, Wroclaw University of Economics and Business, 53-345 Wrocław, Poland; alicja.grzeskowiak@ue.wroc.pl; 4 Semiotic Solutions, Nowa Iwiczna, 05-500 Warsaw, Poland; m.zurawicka@semiotyka.com (M.Ż.); k.polak@semiotyka.com (K.P.)

**Keywords:** attitudes towards people with disability, culture dimensions, analysis of survey results, semiotic analysis

## Abstract

The perception of people with disability (PwD) is of key importance for the full inclusion of this group in the labour market. The article presents selected results of research on the perception of PwD in the workplace. The analyses are based on the results of semiotics research conducted in Poland and of quantitative study in the form of computer-assisted Internet interviews (CAWI) carried out on representative samples from eight European countries. Opinions of Internet users were collected in Belgium, Bulgaria, Greece, Spain, Germany, Poland, Sweden and Great Britain. The results of semiotic analyses on texts mainly from Polish culture made it possible to identify the prevailing images of disability in Polish popular culture and inspired the authors to seek diversity in perceptions of disability depending on social and cultural patterns in a given country. The results of the international survey were used to compare all eight countries with regard to the relationship between the dimensions of culture according to G. Hofstede, and openness to people with disability in the workplace. The conducted research indicates that the perception of the issue of disability is significantly related to the selected dimensions of culture according to G. Hofstede.

## 1. Introduction

Attitudes towards employing people with disability (PwD) have already been studied from many perspectives, both from the point of view of employers [1,2,3,4,5,6,7,8,9,10] and their potential coworkers [11,12,13,14,15,16]. However, browsing various sources it is difficult to find an answer to the question of what really shapes such attitudes of employers and potential coworkers, and how it increases openness towards PwD in the workplace. In our opinion, first and foremost, it is necessary to take into consideration factors present within the enterprise that may be influenced by employers, such as disseminated knowledge and experience in employing PwD, developed organisational culture and infrastructure ensuring adaptation to the needs of PwD. It is necessary to point to the fact that organisational culture is also influenced by factors from outside the enterprise, such as legal solutions and cultural patterns passed on in a given society [17,18,19,20,21,22,23]. On the other hand, openness towards PwD in the workplace meets the attitude displayed by PwD themselves, shaping the experience and future attitudes of employers and coworkers. Among people with disabilities, active (despite limitations) or passive (despite the opportunities in the labour market) attitudes may be influenced by work ethos; that is, cultural patterns of attitudes to professional work passed on in their families, not necessarily corresponding to work ethos in the whole society.

For an in-depth and comprehensive description of social attitudes and behaviour towards people with disability, it is necessary to recognise culture and its influence on the shaping of common social meanings. Semiotics and the tools developed on its basis can play an important role in this process. Paraphrasing the statement of Ferdinand de Saussure, one of the fathers of semiotics, it is possible to state that semiotics is a science analysing the life of signs within social life, which makes it possible to reveal the deep, often unconscious, symbolic meanings and mythologies behind the practices of consumption [24]. Assuming the above, the semiotic analysis of disability reveals unconscious meanings of disability that culture has produced and then disseminated. Their dissemination through cultural texts makes members of a given community assimilate meanings, recognizing them as their own, and then implement them in everyday social practice, behaviour and attitudes—which is manifested, for example, in the approach of entrepreneurs towards employees with disability.

The results of semiotic analyses conducted in December 2018 on texts mainly from Polish culture made it possible to identify the prevailing images of disability. They also inspired the authors to search for diversity in perceptions of disability depending on social and cultural patterns in a given country. Social and organisational culture is most often tested using the dimensions of G. Hofstede [25,26,27]; however, these only indirectly concern the perception of disability in the workplace. The diverse nature of the individual dimensions of culture makes it difficult to define their significance in the context of external factors influencing the effectiveness of activities in the area of diversity management, including disability management. In our research, we decided to apply the solutions proposed by G. Hofstede for several reasons. First of all, in the literature on the subject, G. Hofstede, who started his work in the 1980s, is considered a precursor to research on the dimensions of culture. Of course, it is possible to point to other researchers whose studies focus on various aspects of cultural determinants [28,29,30], but all of them to some extent relate to the dimensions suggested by G. Hofstede. The issue that was of key importance to us was the possibility of free access and use of the quantitative presentation of cultural dimensions for individual countries on a scale from 1 to 100 (see Table A1 in Appendix A). Thanks to such presentation of data, in our comparisons we were able to use a tool appropriate for quantitative methods. It is also worth emphasizing that G. Hofstede indirectly referred to the perception of disability in various societies, especially in the aspect of collectivism–individualism.

**Figure 1:**
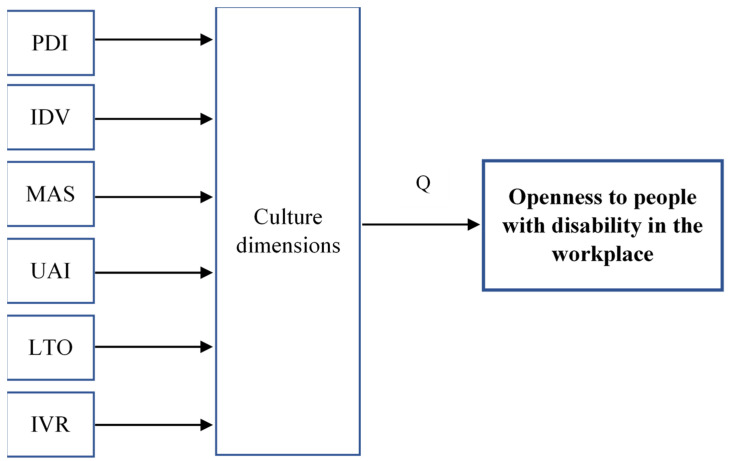
Conceptual model of influence of culture dimensions on the openness towards PwD in the workplace.

Using the answers to the questions from the proprietary questionnaire on openness towards people with disability in the workplace, the conceptual model presented in Figure 1 was built and the following research question was formulated:

How are the dimensions of national cultures defined by G. Hofstede related to the degree of openness towards PwD in the workplace?

Culture dimensions for individual countries are measured by Hofstede using indexes [26,27]. PDI is an index that describes the power distance in a given society. In countries with a significant power distance, social inequalities resulting from the privileges of power are accepted to a much greater extent than in countries with a low PDI value. IDV is an index that measures collectivism vs. individualism of a given society. In individualistic societies, the good of the individual is put before the good of the group. In collectivist societies, it is more important to belong to a “group” that cares for the individual in return for his or her loyalty. MAS is used to measure the femininity vs. the masculinity of society. The low value of the index indicates paying attention mainly to relationships with others, whereas the high value indicates assertiveness, focus on competition and achieving success. UAI measures the perceived level of danger in a given society resulting from ambiguous or unknown situations. The high value of the index indicates significant fears of unconventional behaviour. LTO is an index that measures the normative vs. pragmatic approach. Normative societies display great respect for tradition and suspiciousness towards social changes. IVR is used to measure indulgence vs. restraint of a given society. Low values indicate restraint and controlling behaviour.

In order to find the answer to the research question, we used the results of international comparative studies conducted on representative samples of Internet users from eight European countries. We also conducted an analysis of the relationship at the country level between the dimensions of culture according to Hofstede and openness towards people with disability in the workplace. As auxiliary information, we took into account data on professional activity of people declaring disability as part of the EU-SILC 2018 survey conducted with the use of the standardised Eurostat questionnaire. We performed cross-comparisons aiming at confronting the results from all the eight countries on which the quantitative study was focused.

## 2. Materials and Methods

### 2.1. Data Collection and Research Sample

Analyses are based on the results of the qualitative research conducted in Poland and the quantitative study carried out on representative samples from eight European countries: Belgium, Bulgaria, Germany, Great Britain, Greece, Poland, Spain and Sweden.

Qualitative research was carried out with the use of semiotics and tools developed on its basis. There were analysed texts from the broadly understood area of culture that shape and influence human attitudes and behaviour. Culture-based texts, according to semiotics, are not only written texts but also images, signs, symbols, films, advertising, reportage, etc. In the conducted semiotic research, the texts came mainly from the territory of Poland, while foreign texts were used as auxiliary materials. More than 3000 press texts and 1200 materials from other sources were analysed, including films, online texts and advertisements. The basic source of information were texts from the Polish press from 2016–2018, whereas those issued in the period of 1998–2015 were used as auxiliaries [31].

The quantitative study was in form of computer-assisted Internet interviews (CAWI). Opinions of Internet users aged from 18 to the official retirement age were collected in Belgium (N = 521), Bulgaria (N = 525), Greece (N = 519), Spain (N = 520), Germany (N = 536), Poland (N = 528), Sweden (N = 524) and Great Britain (N = 536). The interviews took place in September–December 2019. The representativeness of the sample in each country was ensured due to such key characteristics as gender, age, education level, place of residence and region of residence. Random-quota sample was used, for which quotas were specified on the base of official Eurostat statistical data concerning populations in the considered countries. Randomness of recruited CAWI participants was ensured by various means of selection, including Internet panels and mobile applications using dynamic sampling. CAWI research was conducted by a specialised survey institute. Respondents gave general consent to data processing and participation in an online panel and took part in the survey voluntarily. The opportunity to opt out was guaranteed anywhere in the survey.

The eventual main sample consisted of 4209 respondents, which were chosen from 4827 complete responses gathered during the survey. The main sample was chosen in order to meet the assumptions about the quotas in each country. Therefore, the analyses were carried out on the basis of this data. It should be underlined that the sample was balanced with respect to gender (female: 49.9%, male: 50.1%) and age (18–34 years old: 32.4%, 35–49 years old: 33.6%, 50–65 years old: 34.0%).

The proprietary questionnaire used in the CAWI survey provided information for evaluation of influence of culture dimensions on the openness towards PwD in the workplace, which is the core idea in our conceptual model. The questions allowing for assessment of the openness towards PwD in the workplace were formulated as follows:

1. Q1: In your opinion, does your country carry out an effective policy that allows for full integration of people with disability?
2. Q2: In your opinion, is there a social atmosphere of understanding the needs and possibilities of people with disability in your country?
3. Q3: Do you think that people with disability who have a job should have special employee privileges, e.g., a shorter working day, longer holidays, etc., in your country?
4. Q4: Do you think that employers in your country have sufficient knowledge of how to employ a person with disability and organise his/her work?

Four various fields of interest are covered by them: the effectiveness of the policy allowing for full integration of PwD, the social atmosphere of understanding the needs and possibilities of PwD, special employee privileges for PwD, the knowledge of employers of how to employ PwD. The answers to the questions were formulated in a 4-point scale, with 1 meaning “definitely no” and 4 meaning “definitely yes”:

A summative scale was also created consisting of assessments of state policy, social atmosphere and the knowledge of employers (questions Q1, Q2 and Q4). Additionally, in order to extend the quantitative analysis, the objective data on employment rates of people with disability in the analysed countries from ANED (EU SILC) dataset [32] were also used. The values of Hofstede’s indexes, average answers obtained from the proprietary questionnaire and the employment rates of people with disability in the countries covered by the study are presented in Table A1 in Appendix A.

### 2.2. Methods of Data Analysis

The understanding of culture is based on the definition by Lotman and Uspensky, i.e., culture is treated as a sign system with an internal hierarchy and its own regularities [33]. This system has a “pattern mechanism”, producing cultural texts having a sign character. From the point of view of this definition, culture is often referred to as a set of texts, although the authors emphasise that it is rather a “mechanism producing a set of texts”. S. Pietraszko defined culture as a system which he described as “the axiosemiotic sphere” [34]. From his point of view, culture is a relatively autonomous creation, ruled by its own mechanisms. In this approach, it is important to distinguish between culture as a system of regularities and manifestations of culture, such as specific cultural texts or human behaviour. In both approaches, the texts of culture are the effects of a certain superior mechanism, a system of regularities manifested in the texts. According to our understanding of culture, social context is a manifestation of the mechanisms of culture, regularities that affect social relations, behaviour and rituals. From this point of view, culture is a mechanism superior to the social context.

The process of identifying the meanings of disability began with the analysis of signs available in popular culture texts, in the press, television, films, literature and music. According to de Saussure’s concept, a sign is a combination of two elements: the signifier: (French *signifiant*, e.g., a word or a picture) and the signified (French *signifié*, e.g., meaning of the word or the picture) [24]. The analysis of signifiers, i.e., physical representations of signs placed in specific cultural contexts, gave a picture of the meanings of disability. Thanks to semiotic research, it was possible to identify threads and topics connected with disability in popular culture, as well as barriers present in the public discourse, so as to better and more effectively communicate the employment of people with disability. The distinguished meanings of the analysed phenomenon, i.e., disability, are organised in broader sequences, the so-called codes, i.e., the most appropriate ways for Polish culture to present and express disability. Codes are often described as a form of cultural acronym; visual, verbal, sound or mixed typical forms of discourse expression (and the culture which this discourse represents) at a specific time in the history of this discourse [35,36]. Evans and Harvey describe the code as a kind of open set of signifiers that relate to one common meaning [37].

**Figure 2:**
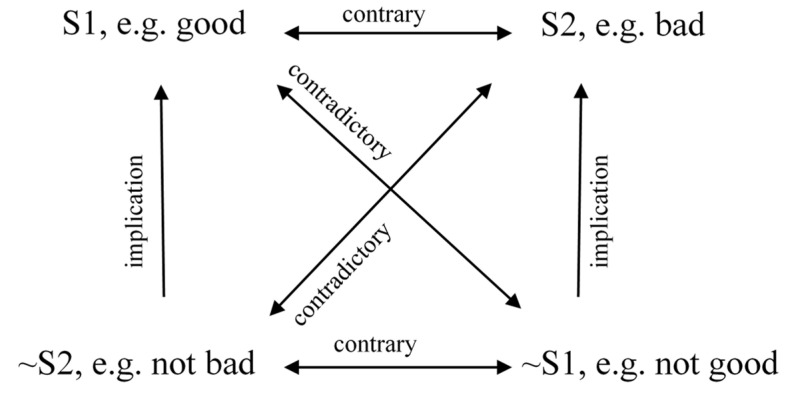
The semiotic square.

Binary oppositions are important in order to understand the basic semantic mechanism, but they do not allow of describing deeper structure of the analysed phenomenon. At this point it is worth using another concept developed on the basis of the Paris school of semiotics: the semiotic square. Created by the Lithuanian semiotician A.J. Greimas [38,39,40], a leading representative of the French school of semiotics, the semiotic square helped to describe and improve many oppositional analyses of such important topics as masculinity–femininity, life–death and good–evil (see Figure 2), precisely by increasing and improving the recognition of analytical classes. The square not only allows for the structuring of the meanings of the analysed phenomenon, but also serves to improve oppositional analyses by increasing the number of analytical classes resulting from a given opposition from two (e.g., life/death) to four (e.g., life/death, non-life/non-death), to eight or even ten [41].

In order to assess the strength of the relationships defined in the conceptual model between the cultural dimensions and the multifaceted construct of openness to people with disability in the workplace, correlation analysis was used. Pearson linear correlation coefficients were calculated and their significance was tested. The most meaningful correlations are shown on scatter plots. The questions reflecting openness were treated both separately and jointly, as three of them constituted a summative scale consisting of assessments of state policy, social atmosphere and the knowledge of employers. The statistical analyses were carried out using Statistica 12.5 [42] and IBM SPSS 26 software [43].

## 3. Results

### 3.1. Semiotic Research

When analysing cultural texts concerning disability, it is possible to notice a number of repetitive signifiers. These include images of a wheelchair, images of people using wheelchairs, images of visually impaired people with a white cane, or images of people with visible motor dysfunctions, different from able-bodied ones. Among these signifiers, it is possible to notice clear prevalence of one of them; there is a symbolic identification of disability with the image of a person in a wheelchair. The occurrence of this sign in public space is caused by cultural texts such as road signs, car parks and TV programmes about people with disability. However, it is worth noting that the same signifier placed in different contexts, according to de Saussure’s sign concept [24], increases the number of meanings. Hence, a different meaning is hidden behind the image of a person in a wheelchair having fun in a go-go club, and a different one in the context of a neglected house in which this person lives. Summarising this disquisition, it is the context that gives meaning to the same signifiers.

The semiotic analysis of disability in the texts from Polish culture allowed us to identify 16 codes organised in sets of signifiers: the world of poverty; depending on others; the weakest and misfits; outstanding and extraordinary; conquer the unconquered; brave warriors; parent—an ordinary hero; individual potential; joy and life energy; me and my passions; one of a kind and unique; the same as the others; typical parents; we live to the fullest; honesty, irony, with a pinch of salt; technology and the improved quality of everyday life. Moreover, it is clearly visible that these codes create a certain specific order based on opposition. On the one hand, we are dealing with codes showing weakness and unhappiness, such as the “the world of poverty” code which identifies disability with living on the breadline, in poverty and even homelessness. This code consists of a set of repetitive signifiers, such as images of neglected and poor people living in extreme conditions and verbal representations from press texts: “locked in poverty”, “50% of the disabled live in poverty”, “people with disability live in fear of lack of money for food”, etc. It is this visual-verbal convention that maintains the belief in Polish society that disability means reliance on others, poverty or unacceptable otherness. On the other hand, the map of codes also includes those which associate disability with exceptionalism, extraordinariness or even courage. Owing to them, disability has gained a new dimension in culture; it is not a weakness anymore, but a sign of heroism and superhuman determination in pursuing one’s goals.

In order to construct a semiotic square for the perception of disability, we relied on the method suggested by J.M. Floch [44], which describes, e.g., the concepts of “good” and “evil”, and in our research we used the terms of “weakness” and “heroism”.

If two people were discussing the notion of weakness and heroism, they would quickly conclude that it is impossible to understand the idea of “weakness” without the idea of “heroism” and the other way round. Both concepts are of relational nature; they create a relational semantic system based on opposition. However, the concepts of “non-weakness” and “non-heroism” may also appear in the discussion, the meanings of which cannot be reduced to the above-mentioned opposite. Discussion participants noted that “non-heroism” is not the same as “weakness” and “non-weakness” is not the same as “heroism”. Between “non-weakness” and “weakness” there is a relationship of contradiction, as there is between “non-heroism” and “heroism”.

Summarising this stage of the research, it is possible to note that disability in basic meaning in Polish culture refers to a simple binary opposition: Heroism vs. Weakness, and both terms are relational.

At this point, it is worth referring to the concept of binary oppositions developed on the basis of semiotics. Jakobson paid attention to the fact that ‘binarism is of key importance; without it the structure of language would be lost’ (as cited in [45]). The representatives of structuralism such as Levi-Strauss also highlighted the role of binary oppositions in understanding and interpretation of culture [46]. Each culture or subculture creates its own sets of oppositions that play a fundamental role in the process of socialisation and bonding of members of a cultural group. In Polish culture, it is the meaning behind the concepts of heroism and weakness, in the context of disability, that determine social perceptions about it, and the most important narratives that bind society together are built around them.

The semiotic square, which reflects the meaning system of weakness and heroism, shows the essence and logic of the square. However, it has its limitations. In this approach, the semiotic square does not explain what stays behind the separated fields of non-weakness and non-heroism. The square reflects the logic of Greimas’s concept, but not the logic of Polish culture concerning disability. In order to accurately describe and understand the actual relations which occur between them, we came back to separated codes (visual-verbal conventions and meanings) so that we were able to answer the question about which of them represent non-weakness and which represent non-heroism.

In the field of non-heroism fit those codes that describe disability as normality associated with ordinary life, where human activities such as sex, love, work or taking care of the family are part of life, as well as the lives of people with disabilities. In cultural texts, disability is shown through the prism of what unites people, not what divides them.

The non-weakness field is the opposite of the concept of normality and stands for empowerment. Disability means power, extraordinariness, originality and a way to distinguish oneself from others. It also makes it possible to enjoy life. The lower opposition of the square extracted in this way, represented by the notions of normality and empowerment, is well-established in western European culture and prevails there, whereas for Polish culture, it is still emerging.

**Figure 3:**
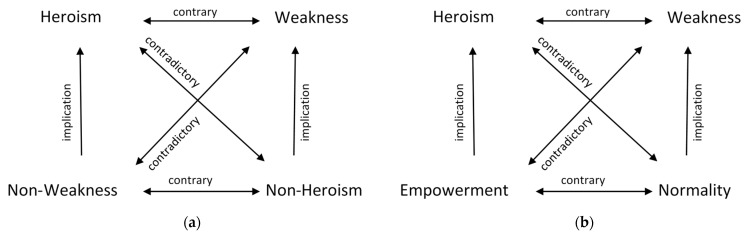
Consequences of semiotic analysis: (**a**) Semiotic square for heroism and weakness; (**b**) Final semiotic square for disability.

Referring to all the presented oppositions and relations between them, we can build the semiotic squares presented in Figure 3.

Summarising the suggested semiotic square for the topic of disability, it is worth revising its logic once again. The fields of weakness and heroism constitute the key binary opposition. Both are of relative nature; they create a relational semantic system based on opposition. The concept of weakness bears all meanings that associate disability with exclusion, humiliation and stigmatisation. In the context of disability, heroism means a challenge and motivation to be stronger and better. The negation of the concept of weakness is the concept of empowerment describing disability as positive otherness, extraordinary and exceptional; the opposite of the concept of heroism is normality. Behind the notion of normality there are meanings associating disability only with one aspect of normal life. The concepts of empowerment and normality create the second semantic system based on opposition.

The role of the defined semiotic square of disability is to explain the mechanism and phenomena occurring in Polish culture. The concept used is the crowning of the entire spectrum of analytical work, i.e., the identification of signs and meanings, distinguishing codes and defining the key oppositions.

According to the square, the obtained order suggests the possibility of abandoning the current methods of communicating disability and the meanings strengthening the stereotypical approach to disability, and changing the situation of people with disability in the labour market and their relationships with employers. Semiotic analysis and the square used in it create new opportunities and point to meanings which are worth disseminating and reinforcing in order to support the assimilation of new and desirable meanings in social behaviour and attitudes.

### 3.2. Influence of Culture Dimensions on the Perception of People with Disability in the Workplace and Their Employability

The adopted conceptual model at least partially confirmed the influence of culture dimensions on the perception and employability of people with disability. In Table 1 we included the values of the Pearson linear correlation coefficients between the Hofstede’s indexes (https://geerthofstede.com/research-and-vsm/dimension-data-matrix/) (accessed on 4 January 2021) representing individual dimensions of culture and average answers to the questions from the proprietary questionnaire, and employment rates of people with disability. The table also shows the results, proving a significant correlation relationship.

Taking into account the dimensions of culture in the context of readiness to employ people with disability, it is necessary to point out the greatest importance of the individualism (IDV) dimension. This is confirmed both by the opinions of respondents and by Eurostat data. Societies with a higher level of individualism offer better legal solutions, create a better social atmosphere of understanding the needs and possibilities of people with disability and ensure better preparation of employers to employ and arrange proper working conditions for people with disability. Consequently, this results in a higher employment rate for this target group. An important dimension of culture is also power distance (PDI). This has a significant influence on the social atmosphere of understanding the needs and possibilities of people with disability and the level of employability of these people. The dimension connected with uncertainty avoidance (UAI) has a major impact on the employment rate of people with disability, while the dimension of indulgence vs. restraint (IVR) determines the acceptability of special employee privileges for people with disability. No statistically significant correlation was found between the answers to the questions from the proprietary questionnaire or the employment rate of people with disability and culture dimensions such as femininity vs. masculinity (MAS) and the normative vs. pragmatic approach (LTO).

**Figure 4:**
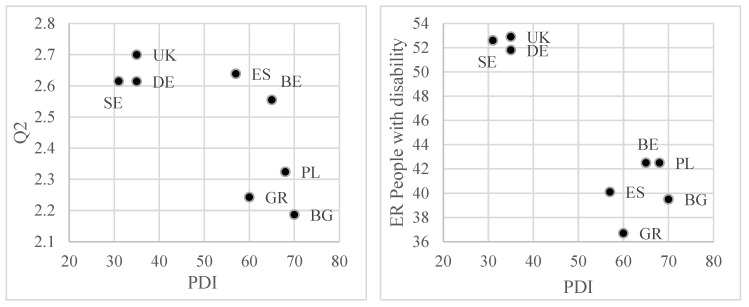

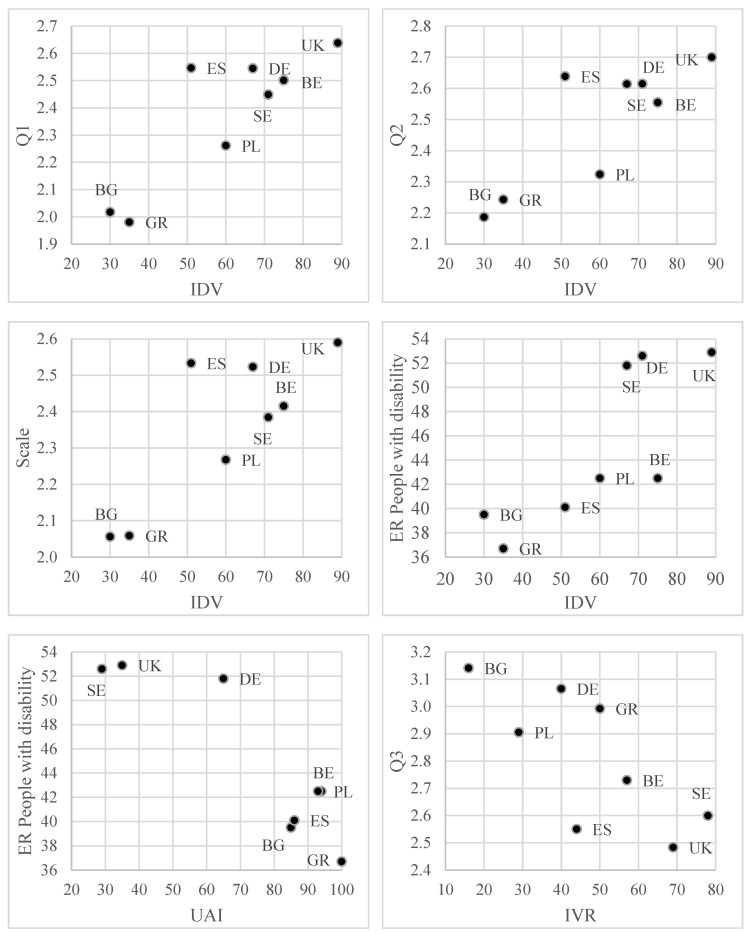
Graphical presentation of the results from Table 1. BG—Bulgaria, DE—Germany, UK—United Kingdom, ES—Spain, BE—Belgium, GR—Greece, PL—Poland, SE—Sweden.

Figure 4 presents correlation graphs for these relationships (pairs of variables and indexes of culture dimensions) that were characterised by a significant relationship for *p*-value < 0.05.

The analysis of the dispersion of points in Figure 4 leads to interesting conclusions. In the case of the power distance dimension and its influence on the employment rates of people with disability, it is possible to distinguish two separate clusters of countries. The first one includes three countries (Sweden, UK, Germany) with relatively low values of the PDI index and, at the same time, a clearly higher level of PwD employment compared to countries from the second cluster. The difference for the average employment rate in both clusters is over 12 pp. In addition, lower power distance translates into a better social atmosphere of understanding the needs and possibilities of people with disability. Belgium and Spain are exceptions, as the social atmosphere was evaluated in a more favourable way than in countries from the second cluster, although the power distance is relatively large. For the dimension of individualism (IDV), the position of two countries (Bulgaria, Greece) differs significantly from the other observations. In these countries, the high level of collectivism translated into low assessments of respondents in the area of openness towards people with disability in the workplace. The highest values of IDV were noted in the UK, with the highest values for the remaining variables included in the chart. An unusual situation occurred for Spain, where, in the absence of any advantage of individualism over collectivism, the evaluation of legal solutions favouring inclusion and an atmosphere of social understanding of PwD was similar to the values for typically individualistic countries. However, it is worth paying attention to the fact that it did not cause any deviation from the general relationship for the level of PwD employment. In the case of the uncertainty avoidance dimension (UAI) and its impact on employability of PwD, two clusters emerged, similarly to PDI. In the first cluster, there are two countries (UK, Sweden) in which there is no tendency to avoid uncertainty, which positively influences openness towards people with disability. The second cluster consists of the remaining countries (except for Germany) and is characterised by a high tendency to avoid uncertainty and a relatively lower employment rate of people with disability compared to the countries from the first cluster. Germany stands out because despite its tendency to avoid uncertainty, the employability of PwD is high compared to the countries from the second cluster. The last of the analysed significant relationships refers to the impact of the dimension of indulgence vs. restraint (IVR) to accept special employee privileges for people with disability. The dispersion of points indicates an inversely proportional relationship, which means that greater restraint favours greater acceptance of privileges by a given society. The highest values were recorded in for Bulgaria, and the lowest for the UK.

## 4. Discussion

International comparative research on representative groups of Internet users was conducted in eight European countries differing in both the level of social and economic development (measured e.g., by GDP per capita PPP, where at the end of 2019 Bulgaria had USD 23,000; Poland had USD 33,100; and Sweden or Germany had over USD 53,000—source: Trading Economics https://tradingeconomics.com/country-list/gdp-per-capita-ppp, accessed on 4 January 2021), as well as solutions in the area of supporting people with disability. Therefore, the results of the research confirming a more positive situation of people with disability in the labour market in countries such as Sweden, the UK or Belgium, compared to the situation in Poland, Greece or Bulgaria, did not come as a surprise. However, some of the outcomes were quite astounding, and the ambiguity of interpretation of answers to the questions for individual countries (e.g., significantly high approval of additional privileges at work for people with disability typical of poorer countries displayed by respondents from Germany) motivated us to focus on cultural differences. The analysis of indexes presenting the dimensions of culture according to Hofstede showed a significant differentiation of the societies covered by the research.

When referring to the obtained results, it is worth pointing to the regularities regarding the identified significant relationships between the dimensions of culture and openness towards PwD. A synthetic presentation of the conclusions is shown in Table 2.

Positive relationships occurred between the individualism dimension (IDV) and many aspects related to openness towards PwD evaluated subjectively by respondents. In highly individualistic countries, there is a better integration policy, a better social atmosphere conducive to the needs of PwD and a high degree of openness. It also translates into an objective indicator showing the situation of PwD in the labour market; the higher the degree of individualism, the higher the employment rate of PwD. A negative correlation was noticed for the power distance (PDI), which is not conducive to openness towards the needs and possibilities of PwD. The power distance dimension is also strongly negatively related to the employment rate, as is the dimension of uncertainty avoidance and anxiety connected with new situations (UAI).

Considering the influence of various dimensions of culture on openness to people with disability, it is worth referring to the analyses carried out by G. Hofstede [26], in which a different attitude to disability in collectivist and individualistic cultures (IDV) is indicated. Based on the results of research conducted among Australian healthcare workers, Hofstede identified different reactions to becoming a person with a disability among different immigrant communities. According to G. Hofstede, in individualistic communities (British, German), a person with a disability maintains the joy of life and optimism and tries to be independent and not use the help of others. In collectivist (e.g., Greek) societies, regret and pessimism dominate, as well as seeking help, usually among family members. In turn, in the case of power distance (PDI) in communities with a smaller distance, greater openness to people with disability can be explained by reducing the social distance between representatives of various social groups and the tendency to treat all members of the community equally, regardless of health problems.

The dimensions of culture proposed by G. Hofstede that we used in our research showed the existence of certain relationships between the characteristics used to describe the community and the perception of disability, including in the workplace. We are aware of the fact that G. Hofstede, who is considered a precursor of research on cultural conditions, probably has as many supporters as opponents. We also know that there are imperfections and a form of tagging or stereotyping of societies related to the proposed dimensions of culture. However, we believe that the benefits resulting primarily from the unified method of their quantitative presentation exceed the possible limitations of G. Hofstede’s approach.

It is worth emphasizing that the research results may have practical implications that can be used in specific workplaces. Knowing the general characteristics of the country in terms of selected dimensions of culture, including, above all, power distance and individualism-collectivism, tailor-made solutions that ensure openness to people with disability in the workplace can be proposed. It seems, however, that regardless of the specific dimensions of culture for a given society, what primarily increases the effectiveness of any action towards full inclusion is knowledge about various types of disability and experience in working with people with disability.

## 5. Conclusions

In the future we are planning to create, on the basis of semiotic study, our own measurement scale [48] of the perception of disability in accordance with four semiotic categories, and then to conduct a quantitative international study using this scale in order to verify and improve it. The development of such a scale will make it possible to compare different countries and analyse the relationship between the results of perception of four disability categories and the dimensions of culture according to G. Hofstede.

It is worth emphasizing that these four semiotic categories were shaped after the systemic transformation in Poland (and other East European countries), under the influence of international patterns in the perception of disability. Therefore, it can be expected that the intensity of the occurrence of similar categories can also be measured in other countries.

Taking into consideration perception of PwD in the workplace, it can be assumed that the perception of disability in the category of normality is conducive not only to the employment but also to the maintenance of employment by PwD. Perception of disability in the category of weakness hinders employment of such people, as with the category of heroism, due to rare experience of benefits resulting from heroism of this type. Perception of disability in terms of empowerment may favour employment, but because of the verification of the employee’s potential in real work it may also contribute to disappointment and reluctance to extend employment.

One should note that the presented results of the correlation analysis have certain limitations. The research was performed only for eight European countries. Verifying results for a larger number of countries could reveal further interesting relationships between culture dimensions and openness towards PwD. It could also influence the evaluation of the significance of the relationships between the analysed variables.

On the other hand, the international comparative study, the results of which are presented in the article, seems to have great cognitive potential and should inspire in-depth research aiming at the explanation of the identified similarities and differences between the analysed countries. It is worth noting that the countries selected for the study differ both in terms of economic development and geographical location, as well as in terms of their historical and religious background. The explanation of the relationship between the indicated features will undoubtedly enrich the research results obtained so far.

## Figures and Tables

**Table 1:** Pearson’s correlation coefficients between culture dimensions according to Hofstede, questions from the proprietary questionnaire and Eurostat data.

Variable	Culture Dimension					
	PDI	IDV	MAS	UAI	LTO	IVR
Q1	−0.619	0.860 *	0.053	−0.553	0.226	0.548
Q2	−0.734 *	0.821 *	−0.078	−0.640	0.179	0.684
Q3	0.428	−0.659	0.260	0.523	0.323	−0.754 *
Q4	−0.455	0.653	0.394	−0.322	0.085	0.202
Scale	−0.628	0.815 *	0.121	−0.529	0.175	0.503
ER People with disability	−0.897 *	0.798 *	−0.124	−0.903 *	0.208	0.582

* *p*-value < 0.05, Scale—summative scale consisting of assessments of state policy, social atmosphere and the knowledge of employers: questions Q1, Q2 and Q4 from proprietary questionnaire, Cronbach’s alpha equal to 0.74 [47], ER People with disability—Employment rate of people with disability, year 2017, ANED [32].

**Table 2:** The significance of culture dimensions in the perception of people with disability in the workplace and in their employment.

Culture Dimension	Variable with Significant Correlation	Direction of Influence	Association
PDI	Q2	(−)	The greater the power distance, the lower the social openness to the needs and possibilities of people with disability.
	ER People with disability	(−)	The greater the power distance, the lower the employment rate of people with disability.
IDV	Q1	(+)	The higher the individualism of society, the more adapted the social policy is to the needs and expectations of people with disability.
	Q2	(+)	The higher the individualism of society, the greater the social openness to the needs and possibilities of people with disability.
	Scale	(+)	The higher the individualism of society, the better the atmosphere and the greater the understanding of the needs and possibilities of people with disability.
	ER People with disability	(+)	The higher the individualism of society, the higher the employment rate of people with disability.
UAI	ER People with disability	(−)	The greater the uncertainty avoidance, the lower flexibility in action and the lower the willingness to employ people with disability.
IVR	Q3	(−)	The greater the indulgence, the lesser the approval of additional privileges for people with disability.

(+) – positive relationship, (−) – negative relationship; ER People with disability—Employment rate of people with disability; Scale—summative scale consisting of assessments of state policy, social atmosphere and the knowledge of employers (questions Q1, Q2 and Q4 from proprietary questionnaire).

## Data Availability

The dataset presented in the study is available upon request from the corresponding author.

## Appendix A

**Table A1:** Values of Hofstede’s indexes, average responses obtained from the proprietary questionnaire and the employment rate of people with disability in specific countries.

Variables	Country							
	BG	DE	UK	ES	BE	GR	PL	SE
PDI	70	35	35	57	65	60	68	31
IDV	30	67	89	51	75	35	60	71
MAS	40	66	66	42	54	57	64	5
UAI	85	65	35	86	94	100	93	29
LTO	69	83	51	48	82	45	38	53
IVR	16	40	69	44	57	50	29	78
Q1	2.02	2.54	2.64	2.55	2.50	1.98	2.26	2.45
Q2	2.19	2.61	2.70	2.64	2.55	2.24	2.32	2.61
Q3	3.14	3.07	2.48	2.55	2.73	2.99	2.91	2.60
Q4	1.96	2.41	2.43	2.42	2.19	1.95	2.22	2.09
Scale	2.06	2.52	2.59	2.53	2.42	2.06	2.27	2.38
ER People with disability	39.5	51.8	52.9	40.1	42.5	36.7	42.5	52.6

BG—Bulgaria, DE—Germany, UK—United Kingdom, ES—Spain, BE—Belgium, GR—Greece, PL—Poland, SE—Sweden, ER People with disability—Employment rate of people with disability, year 2017, ANED [32]. Scale—summative scale consisting of assessments of state policy, social atmosphere and the knowledge of employers: questions Q1, Q2 and Q4 from proprietary questionnaire, Cronbach’s alpha equal to 0.74 [47], Source: https://geerthofstede.com/research-and-vsm/dimension-data-matrix/ (accessed on 4 January 2021), ANED [32] and own preparation.
